# Toxoplasmosis and cytomegalovirus infection and their role in Egyptian autistic children

**DOI:** 10.1007/s00436-023-07818-2

**Published:** 2023-03-14

**Authors:** Zeinab R. Hassan, Kareman M. Zekry, Elham Adel Heikal, Hanan F. Ibrahim, Seham K. Khirala, Samar M. Abd El-Hamid, Doaa R. Amin, Nora Seliem, Gehad N. Abd El-Aal, Mohammad M. Alkherkhisy, Salwa A. Abd Elhamid, Emad A. Mahgoub, Mahmoud E. N. Hefny, Ghada H. El Nady, Mohamed S. Badr

**Affiliations:** 1 Department of Parasitology, Faculty of Medicine for girls, Al-Azhar University, Cairo, Egypt; 2 Department of Medical Microbiology and Immunology, Faculty of Medicine for girls and boys, Al-Azhar University, Cairo, Egypt; 3 Department of Clinical Pathology, Al-Zahraa University Hospital,Faculty of Medicine for girls, Al-Azhar University, Cairo, Egypt; 4 Department of Biochemistry, Faculty of Medicine for girls, Al-Azhar University, Cairo, Egypt; 5 Department of Pediatric, Faculty of Medicine for girls, Al-Azhar University, Cairo, Egypt; 6 Department of Pediatric, Faculty of Medicine, Ain Shams University, Cairo, Egypt; 7 Department of Psychology, Faculty of Art, Cairo University, Cairo, Egypt; 8 Department of Psychological Sciences, Faculty of Education for Early Childhood, Cairo University, Cairo, Egypt; 9 Medical Genetic Center, Department of Medical Genetics, Faculty of Medicine, Ain Shams University, Cairo, Egypt; 10 Medical Research Centre, Department of Molecular Biology, Faculty of Medicine, Ain Shams University, Cairo, Egypt

**Keywords:** Autism, CARS, CMV, ELISA, Prevalence, Toxoplasmosis, RT-PCR

## Abstract

Autism is a neurodevelopmental disorder with a significantly increased incidence rate across the world over the past few years. Toxoplasmosis and cytomegalovirus (CMV) infection are globally prevalent and have been associated with diverse neurological and psychiatric disorders. A few studies have demonstrated the role of toxoplasmosis and CMV as potential etiological factors for autism. Accordingly, this study was performed to estimate the relationship between toxoplasmosis and CMV infection in children with autism as well as to assess their impact on the Childhood Autism Rating Scale (CARS) score. A total of 45 autistic children (6 girls, 39 boys) and 45 (21 girls, 24 boys) healthy control children were enrolled in our study. Their blood samples were collected and tested for the presence of *Toxoplasma* and CMV (IgG and IgM) antibodies and DNA by ELISA and real-time PCR (RT-PCR), respectively. Toxoplasmosis was detected in 11 (24.4%) autistic children through the ELISA [10 (22.2%) IgG + /IgM − and 1 (2.2%) IgG + /IgM +]; however, RT-PCR assay recorded only 1 positive case (2.2%), while it was detected in 10 (22.2%) control children through ELISA [9 (20%) IgG + /IgM − and 1 (2.2%) IgG + /IgM +] and 1 (2.2%) by RT-PCR. On the other hand, CMV infection was detected in all autistic children with 44 (97.8%) testing positive by ELISA [24 (53.3%) IgG + /IgM − , 18 (40%) IgG + /IgM + and 2 (4.4%) IgG − /IgM +] and 25 (55.6%) testing positive by RT-PCR assay. In addition, ELISA assay recorded 43 (95.6%) [19 (42.2%) IgG + /IgM + and 22 (48.9%) IgG + /IgM − and 2 (4.4%) IgG-/IgM +] and RT-PCR recorded 21 (46.7%) positive samples in control children with CMV. No significant difference was noted between autistic and control children for the overall prevalence of *Toxoplasma* or/and CMV infection. Similarly, the CARS score indicated a non-significant difference with *Toxoplasma* or/and CMV infection. Our data does not show an association between autism and toxoplasmosis or/and CMV infection. Nevertheless, considering that autistic children are at a high risk of contracting these infections, further studies with a larger sample size are recommended.

## Introduction

Autism is a behavioral condition that can be classified as a heterogeneous neurodevelopmental disorder that affects approximately 1 in 44 children and can be diagnosed after the second year of life (Maenner et al. 2021).

This disease affects both adults’ and children’s capacities for social interaction, communication, and the capacity to react to specific external stimuli. In addition, the autistic condition is accompanied by difficulty with social imagination and interaction, repetitive behavior patterns, and delayed language development (APA 2013; Siniscalco et al. 2018).

The infectious protozoan *Toxoplasma gondii* (*T. gondii*) attacks warm-blooded animals, including humans, and causes toxoplasmosis, a common global disease (CDC 2013). Patients who are immunocompromised (such as cancer patients, those living with HIV, and organ-transplant recipients) and pregnant women are particularly at risk for toxoplasmosis (Foroutan et al. 2018).

The main transmission modes of the parasite are through the consumption of raw or undercooked meat, contaminated water, and cat feces (CDC 2013). Although no symptoms may appear in adults or children after direct transmission of the parasitic infection, some dangerous effects can arise from the congenital transmission through the placenta of pregnant women to their fetuses, which can affect the central nervous and muscular systems (Abdoli et al. 2014).

A correlation between maternal toxoplasmosis and the risk of having autistic children was reported by Spann et al. (2017). Thus, a relationship between maternal toxoplasmosis and the damage of nuclear and mitochondrial DNA in childhood autism may exist (Al Malki et al. 2021). According to Fond et al. (2013), *T. gondii* tachyzoites may invade the diverse types of brain cells in the cerebellum, which then controls the signal transduction mechanisms and signaling pathways involved in different functions, such as the antimicrobial effector functions, immune cell maturation, and cell apoptosis. Wang et al. (2014) reported a connection between *T. gondii* infections with the endoplasmic reticulum’s stress pathway that induces death in neural stem cells. Some studies have suggested that the parasite plays a role in the biochemical abnormalities and brain morphological findings in autism (Prandota 2011). According to Nayeri et al. (2020), toxoplasmosis is a significant risk factor for autism. In addition, children with autism who had *Toxoplasma* parasites were more aggressive than those without the infection (Hamid et al. 2020).

The largest and most complex member of the human herpes virus family, human cytomegalovirus (CMV), which is also a member of the neurotropic beta-herpes virus family, can infect almost every cell type, including retinal epithelial cells, dermal fibroblasts, monocytes/macrophages, smooth muscle cells, and neurocytes and sustentacular cells of the central nervous system. However, several different cell types produce infectious virions, ranging from extremely few (macrophages) to numerous (extremely high) (fibroblasts). Thus, humans are the only hosts for human CMV, which is particularly specific to each species, but can induce long-lasting neurological complications in 10–15% of asymptomatic human CMV infections (Manicklal et al. 2013).

Autism has been associated with CMV infection, particularly when it occurs in the third trimester of pregnancy, which may raise the chance of autism (Kawatani et al. 2010; Maeyama et al. 2018; Shuid et al. 2021). In a retrospective analysis of the association between CMV infection and autism, Sakamoto et al. (2015) found that children with CMV infection showed a considerably greater incidence of autism than did the controls. Measurement of the CMV-DNA load in dried blood spots allowed the determination of the prevalence of CMV infection in children with autism, particularly in those with intellectual disabilities (Engman et al. 2015; Zhang and Fang 2019).

Therefore, the present study was aimed to evaluating whether there was an association between prior or current *Toxoplasma* and/or CMV infection and autism.

### Patients and methods

The present case–control study was conducted at the Parasitology, Microbiology, Clinical Pathology, Molecular, and Pediatric Departments of Al-Azhar University’s Faculty of Medicine, Ain Shams University Hospital. The study included 45 (6 girls, 39 boys) autistic patients and 45 (21 girls, 24 boys) healthy controls (both aged 3–16 years). Children with autism were recruited from the pediatric and adolescent psychiatry outpatient clinic of the Ain Shams University Hospital and other psychological rehabilitation centers. Healthy children were recruited from the pediatric clinic of the Al-Zahraa University hospital with the condition of having no psychiatric diagnosis. Patients using antibiotics were excluded from the study to avoid the possibility of affecting *Toxoplasma* seropositivity.

### Sample size estimation

Power and Sample Size Program software was used to calculate the sample size (PS). To be able to reject the null hypothesis that this odds ratio equals one with probability (power) 0.8, we enrolled 45 case-patients with 1 matched control/case. Regarding this test of the null hypothesis, the type I error probability was 0.05, with a confidence level of 95%.

### Sample collection

Blood samples were collected from the participants with the consent of their parents. Briefly, 5 mL of the blood sample was drawn from each participant using a fresh syringe for each case. The sample was divided into two separate tubes, one without EDTA for a serological assay and the other with EDTA for a molecular assay. Until further assessments, the sample tubes were stored at 4 °C.

### Qualitative serological assessment

The collected EDTA-free blood samples were centrifuged at 3000 rpm for 10 min, and all separated sera were stored at − 70 °C until the detection test for *Toxoplasma* (IgG and IgM) and CMV (IgG and IgM) separately by using commercially available ELISA kits (Sigma-Aldrich; USA). We used micro-ELISA strip plate wells (separately pre-coated with an antigen specific to *Toxoplasma*-IgG, *Toxoplasma*-IgM, CMV-IgG, or CMV-IgM) that subsequently combined with their unique antigen. Briefly, a wash buffer was diluted with dilution water in the ratio of 1:40. Then, 100 µL of the sample diluent was added to the corresponding well (an empty well served as a blank control, and two empty wells were maintained as negative and positive controls). The sample (10 μL) was added to the corresponding well, and 100 uL of negative and positive controls were added to the negative and positive control wells, respectively. The wells were gently shaken, incubated for 30 min at 37 °C, and then washed with a washing buffer (5 times). The wells were filled with horseradish peroxidase (HRP)-conjugate reagent, incubated, and finally rinsed. In order to develop the color, chromogen solutions were added to the wells and the plates were incubated for 15 min at 37 °C. Then, 50 L of the stop solution was added to each well. Spectrophotometric readings of the plates were taken at 450 nm optical density. Positive *Toxoplasma*-IgG, *Toxoplasma*-IgM, CMV-IgG, or CMV-IgM samples were separately determined through data comparison with their cutoff values, while a negative OD value < cutoff and a positive OD value ≥ cutoff.

### Molecular assessment by RT-PCR

The blood samples obtained from the participants were collected into tubes containing K3 EDTA, and the DNA was extracted from 200 µL of the blood sample by using the Gene JET Whole Blood Genomic DNA Purification Mini Kit (Thermo Scientific) according to the manufacturer’s instructions. The extracted DNA was stored at – 70 °C until further processing. RT-PCR assay was performed on 50 ng of the total extracted DNA using the LightCycler® Multiplex DNA Master (Cat. No. 07 339 585 001) (TIB MOLBIOL, Berlin, Germany). The identification of *Toxoplasma* DNA was performed with specifically designed primers (Thermo Scientific, Brazil) and those specific for the *B1* gene (5′-AACGGGCGAGTAGCACCTGAGGAGA-3′ and 5′-TGGGTCTACGTCGATGGC ATGACAAC-3′) (Evangelista et al. 2021). In each reaction, we processed a negative control (mixture without DNA) and cloned purified *Toxoplasma* DNA available from a previously published qPCR assay (Hanafy et al. 2018) as a positive control. The reaction was performed with the LightCycler (Roche Diagnostics) thermal cycler using the following programming conditions: 15 min at 95 °C for initial denaturation followed by 35 cycles of 30 s at 95 °C, 1 min at 54 °C, and finally 30 s at 72 °C.

CMV was identified using the LightMix® Modular CMV 500 (Cat. No. 50–0130-96) (TIB MOLBIOL, Berlin, Germany), according to the manufacturer’s instructions. Quantitative PCR was performed on the LightCycler (Roche Diagnostics,Mannheim, Germany) thermal cycler using the reaction conditions recommended by the manufacturer. The program conditions were 5 min at 95 °C, followed by 45 cycles as follows: 5 s at 95 °C; 60 °C for 15 s; 72 °C for 15 s then 1 cycle at 40 °C for 30 s. The standard curve was processed using serially diluted DNA in Milli-Q water.

### CARS score assessment

CARS score consists of 15 items that are used to generate a total score to define the severity of autism. A total score of 30–36.5 indicates mild to moderate autism, whereas 37–60 denotes severe autism. CARS are scored by observing the child and interviewing the family. The items in the scale include questions on the relationship to people, imitation, emotional response, body use, object use, adaptation to change, visual response, listening response, perceptive response and usage, fear or anxiety, verbal communication, non-verbal communication, activity level, consistency and level of intellectual response, and finally the general impressions (Rellini et al. 2004; Schopler et al. 2008).

## Statistical analysis

The data were reviewed, assigned codes, and enrolled into the Statistical Package for Social Science (IBM SPSS; version 23). Quantitative data were displayed as means, standard deviations, and ranges. In addition, non-quantitative data were displayed as numbers and percentages. Comparison of the data was performed by chi-square test (χ^2^) for non-quantitative data and by *t*-test and ANOVA for quantitative data. Also, odds ratio (OR) and 95% confidence interval (CI) were estimated. *P* < 0.05* was considered to indicate statistical significance.

## Results

Depending on the current observation, the enrolled children were classified into 4 groups: autistic with *Toxoplasma*, autistic without *Toxoplasma*, control with CMV, and control without CMV.

### Serological and RT-PCR results

The results demonstrated that, out of the total 45 autistic children, 11 (24.4%) (10 boys, 1 girl) were positive for *Toxoplasma* and only 10 (22.2%) (9 boys, 1 girl) out of the 45 control children were positive (Table 1). Autistic children with *Toxoplasma* represented 10 (22.2%) IgG + /IgM − and 1 (2.2%) IgG + /IgM + by ELISA, and 1 (2.2%) positive DNA by RT-PCR (this positive sample was IgG + /IgM + by ELISA) (Table 3). Among the control children with *Toxoplasma*, ELISA recorded 9 (20%) IgG + /IgM − and 1 (2.2%) IgG + /IgM + , albeit RT-PCR detected positive DNA in only 1 (2.2%) case (which was IgG + /IgM + by ELISA) (Table 3). No significant difference was noted between the autistic and control children concerning the overall prevalence of toxoplasmosis (Table 3).

**Table 1:** Age and sex distribution of autistic and control children with or without *Toxoplasma*

Groups	Age	Sex	Total	*χ2*	*P*-value	OR	95% CI (*P* value)
Autistic children	3−16	39 boys (86.7%) 6 girls (13.3%)	45	54.50	*p* < 0.001*	1.13	0.43 to 3.01 (*P* = 0.8)
Autistic with *Toxoplasma*	3−14	10 boys (90.9%) 1 girl (9.1%)	11 (24.4%)				
Autistic without *Toxoplasma*	3−14	29 boys (85.3%) 5 girls (6.8%)	34 (75.6%)				
Control children	3−16	24 boys (53.3%) 21 girls (46.7%)	45	64.07	*p* < 0.001*		
Control with *Toxoplasma*	4−16	9 boys (90%) 1 girl (10%)	10(22.2%)				
Control without *Toxoplasma*	4−16	15 boys (42.9%) 20 girls (57.1%)	35 (77.8%)				

On the other hand, all enrolled autistic children (100%) (39 boys, 6 girls) and 43 (95.6%) (24 boys, 19 girls) of the control children were positive for CMV infection (Table 2). Autistic children with CMV represented 24 (53.3%) IgG + /IgM − , 18 (40%) IgG + /IgM + , and 2 (4.4%) IgG-/IgM + by ELISA, while 25(55.6%) were positive DNA by RT-PCR (including 18 cases were IgG + /IgM + , 4 were IgG + /IgM − , 2 were IgG − /IgM + , and only 1 case was IgG − /IgM −) (Table 3). Among the control children with CMV, there were 22(48.9%) IgG + /IgM − , 19(42.2%) IgG + /IgM + , and 2 (4.4%) IgG-/IgM + , while 21 (46.7%) were positive for CMV-DNA by RT-PCR including (19 were IgG + /IgM + and 2 were IgG − /IgM +) (Table 3). No significant difference was noted between the autistic and control children regarding the overall prevalence of CMV (Table 3).

**Table 2:** Age and sex distribution of autistic and control children with or without CMV

Groups	Age	Sex	Total	*χ2*	*P*-value	OR	95% CI (*P* value)
Autistic children	3−16	39 boys (86.7%) 6 girls (13.3%)	45	187.50	*p* < 0.001*	5.23	0.24−112.07 (*p* = 0.3)
Autistic with CMV	3−16	39 boys (86.4%) 6 girls (13.6%)	45 (100%)				
Autistic without CMV	3−16	0 boy (0%) 0 girls (0%)	0 (0%)				
Control children	3−16	24 boys (53.3%) 21 girls (46.7%)	45	179.09	*p* < 0.001*		
Control with CMV	3−16	24 boys (55.8%) 19 girls (44.2%)	43(95.6%)				
Control without CMV	3−16	0 boys (0%) 2 girls (100%)	2(4.4%)

**Table 3:** Results of ELISA and RT-PCR for detection of *Toxoplasma* or/ and CMV in autistic and control children

Group	*Toxoplasma* (+ve)				CMV (+ve)				*Toxoplasma* and CMV (+ve)	
	ELISA			RT PCR	ELISA			RT PCR	ELISA	RT PCR
	IgG+/IgM+	IgG+/IgM-	IgG-/IgM+	DNA	IgG+/IgM+	IgG+/IgM-	IgG-/IgM+	DNA		
Autistic children	1(2.2%)	10 (22.2%)	0	1(2.2%( (1 with IgG+/IgM+)	18 (40%)	24 (53.3%)	2 (4.4%)	25(55.6%) (18 with IgG+/IgM+, 4 with IgG+/IgM, 2 with IgG-/IgM+ and 1 with IgG-/IgM-)	11 (24.4%)	1 (2.2%)
Boy	1	9	0	1	15	22	0	20		
Girl	0	1	0	0	3	1	2	5		
Total	11(24.4%)				45 (100%)				*χ2*	19.49
									*p* < 0.001*	
Control children	1(2.2%)	9(20%)	0	1(2.2%) (1 with IgG+/IgM+)	19 (42.2%)	22 (48.9%)	2 (4.4%)	21(46.7%) (19 with IgG+/IgM+ and 2 IgG-/IgM+)	10 (22.2%)	0
Boy	0	9	0	0	9	14	1	11		
Girl	1	0	0	1	10	8	1	10		
Total	10(22.2%)				43(95.6%)				*χ2*	22.77
									*p* < 0.001*	
*χ2*	0.04				2.69					
*P* value	*p* > 0.05				*p* > 0.05					

The results indicated a significant difference between ELISA and RT-PCR assays for the detection of *Toxoplasma* and CMV among autistic and control children (Table 3). CMV demonstrated a higher significant prevalence than *Toxoplasma* either in autistic or control children (Table 4). A significant difference was noted in terms of sex distribution among the autistic children with *Toxoplasma* or CMV with a higher incidence in boys than in girls (Table 4). Regarding the age, CMV showed a higher incidence across different ages of all participated children than *Toxoplasma* (Table 5).

**Table 4:** Relationship between *Toxoplasma* and CMV prevalence in autistic and control children

Groups	Age	Sex				Total	*χ2*	*p*-value
		Boys	Girls	*χ2*	*p*-value			
Autistic with *Toxoplasma*	3−14	10 (90.9%)	1 (9.1%)	130.57	*p* < 0.001*	11 (24.4%)	118.35	*p* < 0.001*
Autistic with CMV	3−16	39 (86.4%)	6 (13.6%)	103.10	*p* < 0.001*	45 (100%)		
Control with *Toxoplasma*	3−16	9 (90%)	1 (10%)	124.82	*p* < 0.001*	10 (22.2%)	108.27	*p* < 0.001*
Control with CMV	3−16	24 (55.8%)	19 (44.2%)	2.25	*p* > 0.05	43 (95.6%)

**Table 5:** Different ages of all participated children with or without *Toxoplasma* or CMV infection

Autistic children					Control children			
Age (Years)	Total number	%	*Toxoplasma* (+ve)	CMV (+ve)	Total number	%	*Toxoplasma* (+ve)	CMV (+ve)
3	2	4.4%	1	2	1	2.2%	0	1
4	7	15.6%	5	7	7	15.6%	1	7
5	7	15.6%	1	6	5	11.1%	2	5
6	7	15.6%	0	7	3	6.7%	0	2
7	4	8.9%	0	4	5	11.1%	2	5
8	2	4.4%	1	2	4	8.9%	1	4
9	8	17.8%	1	8	3	6.7%	1	3
10	2	4.4%	0	2	8	17.8%	1	8
11	0	0%	0	0	2	4.4%	1	2
12	3	6.7%	1	3	0	0%	0	0
13	1	2.2%	0	1	3	6.7%	0	3
14	1	2.2%	1	1	1	2.2%	0	1
15	0	0%	0	0	2	4.4%	0	1
16	1	2.2%	0	1	1	2.2%	1	1

### CARS study evaluation

The CARS score in the autistic children with and without *Toxoplasma* or CMV was not significantly different (Table 6). Comparison of CARS scores in the autistic group with *Toxoplasma* revealed a non-significant difference relative to other autistic groups (Table 6).

**Table 6:** Severity of autistic criteria by CARS in different groups

Study group	CAR S score (mean ± SD)	In Relation to CARS score of Autistic with *Toxoplasma*	
		*T*-test	*P* value
Autistic with *Toxoplasma*	36.1 + 10.6		
Autistic without *Toxoplasma*	34.1 + 7.7	0.576	*P* > 0.05
Autistic with CMV	36.7 + 10.65	0.168	*P* > 0.05
Autistic with *Toxoplasma* & CMV	37.6 + 10.3	0.337	*P* > 0.05
ANOVA *test*			
*F*-test	0.606		
*P* value	*P* < 0.05		

## Discussion

Though the underlying causes of autism are not fully understood, many risk factors may be involved, as genetic and environmental factors. Prenatal and postnatal exposure to various pathogens can also be considered as potential risk factors (APA 2013; Siniscalco et al. 2018). There are no previous prospective studies assess the severity of the clinical manifestations of autism associated with congenital toxoplasmosis/CMV versus infection after birth. However, congenital toxoplasmosis/CMV as well as infection at early period of life may cause neuronal damage that can be associated with neuropsychiatric disorders. In addition, some authors reported that children, adolescents, and adult with positive toxoplasmosis or CMV antibodies showed a higher rate of behavior and intellectual disorders compared to normal controls of the same age (Tedla et al. 2011; Burgdorf et al. 2019). The observed association between toxoplasmosis or CMV and behavior changes may be due to increase the level of dopamine that can occur during the infection (McConkey et al. 2013; Pandey et al. 2014). Also, in autism, there is an increase in the level of dopamine (Nakamura et al. 2010) and these similarities supported that congenital and chronic latent toxoplasmosis or CMV infection can result in the clinical manifestations of autism as some authors reported (Nayeri et al. 2020; Shuid et al. 2021).

In the present study, the incidence of toxoplasmosis among autistic children was 24.4% (11 participants) when compared to 22.2% (10 participants) in the control children, and the difference was not statistically significant. This finding is consistent with previous reports of a non-significant correlation between autism and *Toxoplasma* infection in Iran (Afsharpaiman et al. 2014), Turkey (Esnafoglu et al. 2017), and Egypt (Gouda and Shafey 2020). In contrast, other research demonstrated a significant relationship between autism and *Toxoplasma* infection in Egypt (Prandota et al. 2015), Saudi Arabia (AL Malki et al. 2021), and Iraq (Bazzaz and Jameel 2022), which was also supported by a meta-analysis reported from different countries (Nayeri et al. 2020). The low prevalence of toxoplasmosis in autistic children in the current study may be attributed to the use of antipsychotic treatment in almost all patients. In support, Leweke et al. (2004) elucidated that the use of antipsychotic treatment in schizophrenic patients reduced antibody levels. In addition, Fond et al. (2014) and Frye et al. (2019) noticed an in vitro inhibition of *Toxoplasma* replication with antipsychotic drugs. Prandota et al. (2015) suggested that toxoplasmosis can exhaust the immune response and reduce the B-lymphocyte activation, which in turn reduces the levels of IgG and IgM, making serum *Toxoplasma*-IgG and IgM negativity a point of debate.

The current ELISA assay for toxoplasmosis in autistic children detected 11 (24.4%) of the antibodies [10 (22.2%) IgG + /IgM − and 1 (2.2%) IgG + /IgM +], while only 1 case (2.2%) of the *Toxoplasma* DNA was detected by RT-PCR assay. In control children with *Toxoplasma*, ELISA recorded 10 (22.2%) [9(20%) IgG + /IgM − and 1 (2.2%) IgG + /IgM +], while RT-PCR recorded only 1 (2.2%) case. A significant difference between the serological and molecular results thus confirmed chronic (latent) toxoplasmosis for most cases, with only 1 case showing acute or reactive infection in both autistic and control children. These results conform to those of Zainodini et al. (2013), Ibrahim et al. (2017), and Elzeky et al. (2022), who reported that *Toxoplasma*-IgG assay showed higher prevalence than RT-PCR assay for *Toxoplasma* gene, but that RT-PCR was more reliable and sensitive to detect reactivated or acute infections than *Toxoplasma*-IgM assay.

In terms of the age of the participants, toxoplasmosis revealed no relation between seropositivity and age, as also supported by Zhou et al. (2019) and Elzeky et al. (2022). The age of seropositivity cases ranged from 3 to 14 years in autistic children, while it was 3–16 years in control children, and the absence of seropositivity in older autistic children may be attributed to the longer period of antipsychotic administration, as described elsewhere (Fond et al. 2014; Frye et al. 2019).

However, there was a higher significant *Toxoplasma* seropositivity in boys than in girls in both the autistic and control groups, which was similar to sex distribution in past studies (Prandota et al. 2015; Esnafoglu et al. 2017). However, Gouda and Shafey (2020) have reported that autistic girls were more affected by *Toxoplasma* than boys, which can be explained by several reasons. For instance, according to Kang et al. (2004), the immune response differs across gender in adolescents. Otherwise, Kankova et al. (2007) reported that mothers with toxoplasmosis had affected male offspring more. Moreover, in the present study, the number of participating boys was higher than girls, especially in autistic children, which may be an additional cause for this significant gender seropositivity difference, and the lower number of autistic female children enrolled in the study can be explained by the results of Windham et al. (2013), who suggested that girls had a lower genetic risk for autism.

On the other hand, the incidence of CMV among autistic children and control children was 100% (45 participants) and 95.6% (43 participants), respectively, with a non-significant difference. Similarly, Gentile et al. (2014) and Valayi et al. (2017) reported a high level of CMV seropositivity in autistic children and healthy controls with non-statistically significant differences between both groups. In contrast, other researchers indicated that autistic cases had a significantly higher rate of CMV prevalence than healthy children (Kawashti et al. 2006; Engman et al. 2015; Sakamoto et al. 2015). In the current study, CMV showed a high prevalence in both autistic and control groups, which conforms with previous reports suggesting a high prevalence of CMV seropositivity, especially in developing countries (Lachmann et al. 2018; Arapović et al. 2020; Kahraman and Savcı 2022).

For anti-CMV antibodies, 44 (97.8) autistic children were found positive by ELISA [24 (53.3%) IgG + /IgM − , 18 (40%) IgG + /IgM + , and 2 (4.4%) IgG − /IgM +], while 25 (55.6%) were found positive by RT-PCR for CMV-DNA. In control children with CMV, ELISA recorded 43 (95.6%) [19 (42.2%) IgG + /IgM + , 22 (48.9%) IgG + /IgM − , and 2 (4.4%) IgG − /IgM +] and RT-PCR recorded 21 (46.7%) cases. Seropositivity of CMV antibodies was significantly higher by ELISA than by RT-PCR, which agrees with some data reported previously (El Sanousi et al. 2016; Arora et al. 2018). Our observation suggests that almost half of the enrolled autistic and control children had latent CMV infection with reactivation or active infection. Although congenital CMV infection had been implicated with a variety of neural deficits including autism, our results could not confirm this etiological relevance that was masked by the high prevalence in all participants.

Furthermore, CMV infection in both autistic and control children showed a high incidence across different ages, as also reported previously by Arora et al. (2018). In terms of gender, a significant difference was noted in CMV prevalence between boys and girls, with a higher rate in boys than in girls; however, it should be considered that the enrolled autistic children had more numbers of girls than boys. Also, considering that most of the children from both sexes were affected, we cannot consider this data as the absolute significance and can consider it a bias. Meanwhile, a similar result has been observed by Seale et al. (2006), Arora et al. (2018), and Franjića et al. (2020). In contrast, Adeiza et al. (2016) mentioned higher rates of CMV-IgM seroprevalence in males, while Shukla et al. (2015) and Bakri et al. (2016) reported a higher incidence of CMV-IgG seroprevalence among females.

Interestingly, both autistic and control children showed significantly higher CMV incidence than toxoplasmosis, which conforms to the reports of Mahic et al. (2017), Frye et al. (2019), and Kahraman and Savcı (2022). Despite the absence of any significant difference in the prevalence of *Toxoplasma* or CMV between the autistic and control groups, the autistic children documented a higher risk of infection than the control, which can be explained by the association with immune abnormalities in autistic children compared to the healthy ones, as reported previously (Raouf et al. 2022).

The present study demonstrated a non-significant difference in CARS score between all intra-autistic groups (with and without *Toxoplasma* and/or CMV infection), as well as no difference in autistic children with *Toxoplasma* when compared to others with CMV or/and *Toxoplasma*. Although the highest mean CARS score was recorded in autistic children with combined CMV and *Toxoplasma* infection followed by CMV alone and then *Toxoplasma* alone, the lowest score was recorded in autistic children without any of these infections*.* Similarly, Prandota et al. (2015) observed no marked difference in the CARS value in autistic children with and without *Toxoplasma*, attributable to a difference in the age of the participants and their immune system maturation. Similarly, Slawinski et al. (2018) demonstrated that maternal CMV seropositivity had no association with the severity of the Social Responsiveness Scale, Second Edition (SRS-2) scores in autistic children. In contrast, Hamid et al. (2020) reported more aggressiveness in autistic children with *Toxoplasma* than in those without it. Furthermore, Gentile et al. (2014) noticed that autistic children with CMV seropositivity exhibited more severity behavior scales when compared to those without.

## Conclusions

The present results suggested that the rate of prevalence of toxoplasmosis or/and CMV infection has a non-significant difference between autistic and control children. Nevertheless, autistic children can be considered a high-risk group for both infections. Thus, according to these results, it can be concluded that either toxoplasmosis or CMV infection does not seem to play a role in the etiology of autism among Egyptian children. One of the limitations of the current study is that it does not differentiate between the impacts of congenital infection versus later infection in life on the severity of autism. Further studies with greater numbers of contributors with diverse age groups and different study designs are warranted to obtain more decisive results.

## Data Availability

The corresponding author will reply to reasonable requests for the datasets used and/or analyzed during the current work.

## References

[CR1] Abdoli A, Dalimi A, Arbabi M, Ghaffarifar F (2014). Neuropsychiatric manifestations of latent toxoplasmosis on mothers and their offspring. J Matern-Fetal Neo.

[CR2] Adeiza MA, Dalhat MM, Musa BOP, Muktar HM, Garko SB, Habib AG (2016). Seroepidemiology of cytomegalovirus antibodies in HIV-positive and HIV-negative adults in Nigeria. Sub Saharan Afr J Med.

[CR3] Afsharpaiman S, Skandari A, Maryam ZJ, Radfar S, Shirbazoo S, Amirsalari S, Torkaman M (2014). An assessment of Toxoplasmosis antibodies seropositivity in children suffering autism. Tehran Univ Med J.

[CR4] Al Malki JS, Hussien NA, Al Malki F (2021). Maternal toxoplasmosis and the risk of childhood autism: Serological and molecular small-scale studies. BMC Pediatr.

[CR5] APA (2013) Diagnostic and Statistical Manual of Mental Disorders. (fifth ed.). American Psychiatric Publishing, Arlington, VA (2013)

[CR6] Arapović J, Rajič B, Pati S, Brizić I, Azinović I, Šušak B, Ostojić M, Tutiš B, Raguž AB, Tomić V, Jonjić S, Boppana S (2020). Cytomegalovirus seroprevalence and birth prevalence of congenital CMV infection in Bosnia and Herzegovina: a single-center experience. Pediatr Infect Dis J.

[CR7] Arora T, Devi P, Singh K, Kaur Sidhu S, Oberoi L, Sita Malhotra A (2018). Detection and quantification of cytomegalovirus in immunocompromised patients. Int J Contem Med Res.

[CR8] Bakri MM, Agag A, Alnemri A, Hoban Yi NA, Alaamri AI, Eisa Z (2016). Serostatus of cytomegalovirus among population, Jazan region. Saudi Arabia Sky J Microbiol Res.

[CR9] Bazzaz AA, Jameel JM (2022). Prevalence of the parasite Toxoplasma gondii in Autistic Children in Iraq. Clin Immunol Res.

[CR10] Burgdorf KS, Trabjerg BB, Pedersen MG, Nissen J, Banasik K, Pedersen OB, Sørensen E, Nielsen KR, Larsen MH, Erikstrup C, Bruun-Rasmussen P, Westergaard D, Thørner LW, Hjalgrim H, Paarup HM, Brunak S, Pedersen CB, Torrey EF, Werge T, Mortensen PB, Yolken RH, Ullum H (2019). Large-scale study of Toxoplasma and Cytomegalovirus shows an association between infection and serious psychiatric disorders. Brain Behav Immun.

[CR11] Center of Disease Control CDC (2013) Parasites Toxoplasmosis (Toxoplasma infection) [Online]; 1600 Clifton Road, Atlanta, GA30333: Centers for Disease Control and Prevention. Available online https://www.cdc.gov/parasites/toxoplasmosis/index.html. Accessed 5 Sep 2022

[CR12] El Sanousi MS, Osman ZA, Mohamed ABS, Awfi M, Babair HY, Babair HM (2016). Comparison of real-time PCR versus ELISA in the diagnosis of cytomegalovirus infection in pregnant women. Clin Microbiol Infect Dis.

[CR13] Elzeky SM, Nabih N, Abdel-Magied AA, Abdelmagid DS, Handoussa AE, Hamouda MM (2022) Seroprevalence and genetic characterization of Toxoplasma gondii among children with neurodevelopmental disorders in Egypt. J Trop Med 2022:2343679. 10.1155/2022/2343679 10.1155/2022/2343679 PMC9166983 35669051

[CR14] Engman ML, Sundin M, Miniscalco C, Westerlund J, Lewensohn-Fuchs I, Gillberg C, Fernell E (2015). Prenatal acquired cytomegalovirus infection should be considered in children with autism. Acta Paediatr.

[CR15] Esnafoglu E, Yancar Demir E, Cetinkol Y, Kerem Calgin MK, Erdil A, Yurdakul Erturk E, Dağlı A (2017). The seroprevalence of antibodies to Toxoplasma gondii among children with autism. Düşünen Adam J Psychiatry Neurol Sci.

[CR16] Evangelista F, Ferreira W, Mantelo F, Beletini L, de Souza A, Laet Sant’Ana P, de Lima K, Crestani C, Guilherme A (2021) Rosuvastatin revert memory impairment and anxiogenic-like effect in mice infected with the chronic ME-49 strain of Toxoplasma gondii. PLoS One 16(4):e0250079. 10.1371/journal.pone.0250079 10.1371/journal.pone.0250079 PMC8049280 33857221

[CR17] Fond G, Macgregor A, Tamouza R, Hamdani N, Meary A, Leboyer M, Dubremetz JF (2014). Comparative analysis of anti-toxoplasmic activity of antipsychotic drugs and valproate. Eur Arch Psychiatry Clin Neurosci.

[CR18] Fond G, Capdevielle D, Macgregor A, Attal J, Larue A, Brittner M, Ducasse D, Boulenger JP (2013). Toxoplasma gondii: a potential role in the genesis of psychiatric disorders. Encéphale.

[CR19] Foroutan M, Rostami A, Majidiani H, Riahi SM, Khazaei S, Badri M, Yousefi E (2018). A systematic review and meta-analysis of the prevalence of toxoplasmosis in hemodialysis patients in Iran. Epidemiol Health.

[CR20] Franjić D, Karlović H, Rajič BB, Azinović I, Komšić M, Mikulić V, Šušak B, Miličević T, Arapović M, Bilinovac Ž, Mikulić I, Arapović J (2020). The cytomegalovirus seroprevalence among children in Mostar, Bosnia and Herzegovina: A hospital cross-sectional study. Clin Epidemiol Glob Health.

[CR21] Frye MA, Coombes BJ, McElroy SL, Jones-Brando L, Bond DJ, Veldic M, Romo-Nava F, Bobo WV, Singh B, Colby C, Skime MK, Biernacka JM, Yolken R (2019). Association of cytomegalovirus and Toxoplasma gondii antibody titers with bipolar disorder. JAMA Psychiat.

[CR22] Gentile I, Zappulo E, Bonavolta R, Maresca R, Messana T, Buonomo AR, Portella G, Sorrentino R, Settimi A, Pascotto A, Borgia G, Bravaccio C (2014). Prevalence and titre of antibodies to cytomegalovirus and Epstein-Barr virus in patients with autism spectrum disorder. In Vivo.

[CR23] Gouda MA, Shafey D (2020). Detection of Anti Toxoplasma antibodies in children with autism in Shebin Al-Kom district Menoufia Governorate. Egypt Egypt J Med Microbiol.

[CR24] Hanafy NA, Badr MS, Nasr GM (2018). Performance of 2 polymerization protocols targeting cloned toxoplasma parasites. Open Access Maced J Med Sci.

[CR25] Hamid N, Azizy B, Hamidynejat H (2020). Comparison of the infection of Toxoplasma Gondii and aggression in autism and normal children. SadraSci J.

[CR26] Ibrahim HM, Mohamed AH, El-Sharaawy AA, El-Shqanqery HE (2017). Molecular and serological prevalence of *Toxoplasma gondii* in pregnant women and sheep in Egypt. Asian Pac J Trop Med.

[CR27] Kahraman H, Savcı Ü (2022). Rubella, cytomegalovirus and toxoplasmosis seroprevalence in pregnants in Çorum province. Anatol Curr Med J.

[CR28] Kang DH, Kim CJ, Suh Y (2004). Sex differences in immune responses and immune reactivity to stress in adolescents. Biol Res Nurs.

[CR29] Kanková S, Sulc J, Nouzová K, Fajfrlík K, Frynta D, Flegr J (2007). Women infected with parasite Toxoplasma have more sons. Naturwissenschaften.

[CR30] Kawashti MI, Amin OR, Rowehy NG (2006). Possible immunological disorders in autism: Concomitant autoimmunity and immune tolerance. Egypt J Immunol.

[CR31] Kawatani M, Nakai A, Okuno T, Kobata R, Moriuchi M, Moriuchi H, Tsukahara H, Mayumi M (2010). Detection of cytomegalovirus in preserved umbilical cord from a boy with autistic disorder. Pediatr Int.

[CR32] Lachmann R, Loenenbach A, Waterboer T, Brenner N, Pawlita M, Michel A, Thamm M, Poethko-Müller C, Wichmann O, Wiese-Posselt M (2018). Cytomegalovirus (CMV) seroprevalence in the adult population of Germany. PLOS ONE.

[CR33] Leweke FM, Gerth CW, Koethe D, Klosterkötter J, Ruslanova I, Krivogorsky B, Torrey EF, Yolken RH (2004). Antibodies to infectious agents in individuals with recent onset schizophrenia. Eur Arch Psychiatry Clin Neurosci.

[CR34] Maenner MJ, Shaw KA, Bakian AV, Bilder DA, Durkin MS, Esler A, Furnier SM, Hallas L, Hall-Lande J, Hudson A, Hughes MM, Patrick M, Pierce K, Poynter JN, Salinas A, Shenouda J, Vehorn A, Warren Z, Constantino JN, DiRienzo M, Fitzgerald RT, Grzybowski A, Spivey MH, Pettygrove S, Zahorodny W, Ali A, Andrews JG, Baroud T, Gutierrez J, Hewitt A, Lee LC, Lopez M, Mancilla KC, McArthur D, Schwenk YD, Washington A, Williams S, Cogswell ME (2018). (2021): Prevalence and characteristics of autism spectrum disorder among children aged 8 years—autism and developmental disabilities monitoring network, 11 sites, United States. MMWR Surveill Summ.

[CR35] Maeyama K, Tomioka K, Nagase H, Yoshioka M, Takagi Y, Kato T, Mizobuchi M, Kitayama S, Takada S, Nagai M, Sakakibara N, Nishiyama M, Taniguchi-Ikeda M, Morioka I, Iijima K, Nishimura N (2018). Congenital cytomegalovirus infection in children with autism spectrum disorder: systematic review and meta-analysis. J Autism Dev Disord.

[CR36] Mahic M, Mjaaland S, Bøvelstad HM, Gunnes N, Susser E, Bresnahan M, Øyen A-S,Levin B, Che X, Hirtz D, Reichborn-Kjennerud T, Schjølberg S, Roth C, Magnus P, Stoltenberg C, Surén P, Hornig M, Lipkin WI (2017) Maternal immunoreactivity to herpes simplex virus 2 and risk of autism spectrum disorder in male offspring. mSphere 2(1):e00016–e00017. 10.1128/mSphere.00016-17

[CR37] Manicklal S, Emery VC, Lazzarotto T, Boppana SB, Gupta RK (2013). The “silent” global burden of congenital cytomegalovirus. Clin Microbiol Rev.

[CR38] Nakamura K, Sekine Y, Ouchi Y, Tsujii M, Yoshikawa E, Futatsubashi M, Tsuchiya KJ, Sugihara G, Iwata Y, Suzuki K, Matsuzaki H, Suda S, Sugiyama T, Takei N, Mori N (2010). Brain serotonin and dopamine transporter bindings in adults with high-functioning autism. Arch Gen Psychiatry.

[CR39] Nayeri T, Sarvi S, Moosazadeh M, Hosseininejad Z, Sharif M, Amouei A, Daryani A (2020). Relationship between toxoplasmosis and autism: a systematic review and meta-analysis. Microb Pathog.

[CR40] McConkey GA, Martin HL, Bristow GC, Webster JP (2013). Toxoplasma gondii infection and behaviour – location, location, location?. J Exp Biol.

[CR41] Pandey GN, Rizavi HS, Ren X, Bhaumik R, Dwivedi Y (2014). Toll-like receptors in the depressed and suicide brain. J Psychiatry Res.

[CR42] Prandota J, Abdel Fattah Elleboudy N, Ahmed Ismail K, Kamal Zaki O, Hussein Shehata H (2015) Increased seroprevalence of chronic toxoplasmosis in autistic children: special reference to the pathophysiology of IFN-gamma and NO overproduction. Int J Neurol 1(3):102–122. 10.13140/RG.2.1.1077.1687

[CR43] Prandota J (2011). Metabolic, immune, epigenetic, endocrine and phenotypic abnormalities found in individuals with autism spectrum disorders, Down syndrome and Alzheimer disease may be caused by congenital and/or acquired chronic cerebral toxoplasmosis. Res Autism Spectr Disord.

[CR44] Raouf HA, Kholoussi N, Kholoussi S, Abo-Shanab AM, Ashaat EA, Ashaat NA, Helwa I (2022) Association of immune abnormalities with symptom severity in Egyptian autistic children. Egypt Pharmaceut J 21(2):242–248. Available online http://www.epj.eg.net/text.asp?2022/21/2/242/351586. Accessed 10 Sep 2022

[CR45] Rellini E, Tortolani D, Trillo S, Carbone S, Montecchi F (2004). Childhood Autism Rating Scale (CARS) and Autism Behavior Checklist (ABC) correspondence and conflicts with DSM-IV criteria in diagnosis of autism. J Autism Dev Disord.

[CR46] Sakamoto A, Moriuchi H, Matsuzaki J, Motoyama K, Moriuchi M (2015). Retrospective diagnosis of congenital cytomegalovirus infection in children with autism spectrum disorder but no other major neurologic deficit. Brain Dev.

[CR47] Schopler E, Reichler R, Renner B (2008) The Childhood Autism Rating Scale (CARS). (Twelfth printing).WPS: Los Angeles

[CR48] Seale H, Maclntyre CR, Gidding HF, Backhouse JL, Dwyer DE, Gilbert L (2006). National serosurvey of cytomegalovirus in Australia. Clin Vaccine Immunol.

[CR49] Shuid AN, Jayusman PA, Shuid N, Ismail J, Kamal Nor N, Mohamed IN (2021). Association between viral infections and risk of autistic disorder: an overview. Int J Environ Res Public Health.

[CR50] Shukla S, Singh P, Tidke P, Bhatia V, Dutt S (2015). Prevalence of HCMV in Indian Scenario. IJPRR.

[CR51] Siniscalco D, Schultz S, Brigida AL, Antonucci N (2018). Inflammation and neuro-immune dysregulations in autism spectrum disorders. Pharmaceuticals.

[CR52] Slawinski BL, Talge N, Ingersoll B, Smith A, Glazier A, Kerver J, Paneth N, Racicot K (2018). Maternal cytomegalovirus sero-positivity and autism symptoms in children. Am J Reprod Immunol.

[CR53] Spann MN, Sourander A, Surcel HM, Hinkka-Yli-Salomäki S, Brown AS (2017). Prenatal toxoplasmosis antibody and childhood autism. Autism Res.

[CR54] Tedla Y, Shibre T, Ali O, Tadele G, Woldeamanuel Y, Asrat D, Aseffa A, Mihret W, Abebe M, Alem A, Medhin G, Habte A (2011). Serum antibodies to Toxoplasma gondii and Herpesvidae family viruses in individuals with schizophrenia and bipolar disorder: a case-control study. Ethiop Med J.

[CR55] Valayi S, Eftekharian MM, Taheri M, Alikhani MY (2017). Evaluation of antibodies to cytomegalovirus and Epstein-Barr virus in patients with autism spectrum disorder. Hum Antibodies.

[CR56] Wang T, Zhou J, Gan X, Wang H, Ding X, Chen L, Wang Y, DU J, Shen J, Yu L,  (2014). Toxoplasma gondii induce apoptosis of neural stem cells via endoplasmic reticulum stress pathway. Parasitology.

[CR57] Windham GC, Sumner A, Li SX, Anderson M, Katz E, Croen LA, Grether JK (2013). Use of birth certificates to examine maternal occupational exposures and autism spectrum disorders in offspring. Autism Res.

[CR58] Zainodini N, Zare-Bidaki M, Abdollahi SH, Afrooz M, Ziaali N, Ebrahimian M (2013). Kazemi Arababadi M (2014) Molecular and serological detection of acute and latent toxoplasmosis using real-time PCR and ELISA techniques in blood donors of rafsanjan city, iran. Iran J Parasitol.

[CR59] Zhang XY, Fang F (2019). Congenital human cytomegalovirus infection and neurologic diseases in newborns. Chin Med J (engl).

[CR60] Zhou N, Fu H, Wang Z, Shi H, Yu Y, Qu T, Wang L, Zhang X, Wang L (2019). Seroprevalence and risk factors of Toxoplasma gondii infection in children with leukemia in Shandong Province, Eastern China: a case-control prospective study. PeerJ.

